# Regulation of Arginine Metabolism and Ethanol Tolerance in *Saccharomyces cerevisiae* by *BTN2*

**DOI:** 10.1002/fsn3.70244

**Published:** 2025-05-01

**Authors:** Ting Xia, Keiwei Chen, Huqi Zhou, Tangchao Chen, Wenjing Lin, Gongnian Xiao, Ruosi Fang

**Affiliations:** ^1^ School of Biological and Chemical Engineering Zhejiang University of Science and Technology Hangzhou China; ^2^ Youxian Shop (Zhejiang) Food Co. Ltd. Huzhou China

**Keywords:** arginine metabolism, *BTN2*, ethanol tolerance, *Saccharomyces cerevisiae*

## Abstract

Ethyl carbamate (EC), primarily formed by the reaction between urea and ethanol, is a natural carcinogen prevalent in fermented alcoholic beverages. Urea is an arginine metabolite produced by *Saccharomyces cerevisiae* . Previous studies have shown that *BTN2* influences arginine metabolism. In this study, we compared the effects of *BTN2‐*modified strains on key metabolites, enzymes, and transcriptional gene expressions in the arginine metabolic pathway, and assessed cell growth and oxidative damage under different ethanol stresses. It revealed that the knockout of *BTN2* inhibited arginine intake and promoted urea reduction. RT‐qPCR results demonstrated that *BTN2* regulate arginine transportation, catabolism, and urea degradation by modulating the expression of *GAP1*, *CAN1*, *CAR1*, and *DUR1,2*. Moreover, the results showed that *BTN2* enhanced ethanol tolerance and alleviated cellular damage. These findings provide a promising method for reducing arginine uptake by *Saccharomyces cerevisiae* and consequently urea accumulation in wine.

## Graphical abstract

The impact of *BTN2* modified strains on key metabolites, enzymes, and gene expression in the arginine metabolic pathway was investigated. *BTN2* knock out strain was found to reduce arginine uptake and promote arginine and urea degradation. *BTN2* was found to enhance ethanol tolerance and reduce ethanol‐induced oxidative damage.

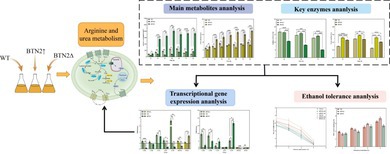

## 1 Introduction

Due to its distinctive flavor and excellent nutritional value, Chinese yellow rice wine is popular among local and foreign consumers (Jiao et al. 2014). However, it contains several hazardous compounds, notably ethyl carbamate (EC) (Chen et al. 2022; Wang et al. 2021). EC is produced through a chemical reaction between ethanol and carbamoylated compounds such as urea, citrulline, carbamoyl phosphate, cyanide, and diethyl pyrocarbonate, with urea serving as the main precursor. Urea mainly originates from raw materials and through the accumulation of arginine metabolites in *Saccharomyces cerevisiae* (Gowd et al. 2018). Therefore, it is of great importance to inhibit urea formation during fermentation. Previous studies have explored approaches to reduce urea production in *S. cerevisiae* using genetic modification technology with a focus on two strategies: knocking out the arginase gene (Wu et al. 2014; Guo et al. 2016) or increasing the expression of urea‐degrading enzyme and transporter proteins genes (Zhang et al. 2017; Wu, Xie, et al. 2020). Although these approaches effectively reduced urea content, the utilization pathway of arginine was disrupted, which led to arginine accumulation in the fermentation broth, ultimately impacting the flavor and quality of the product (Zhao et al. 2013). Therefore, in recent years, researchers have focused on the arginine transport pathway to control the entry of arginine into yeast (Zhang et al. 2016; Zhang and Hu 2018).

**FIGURE 1:**
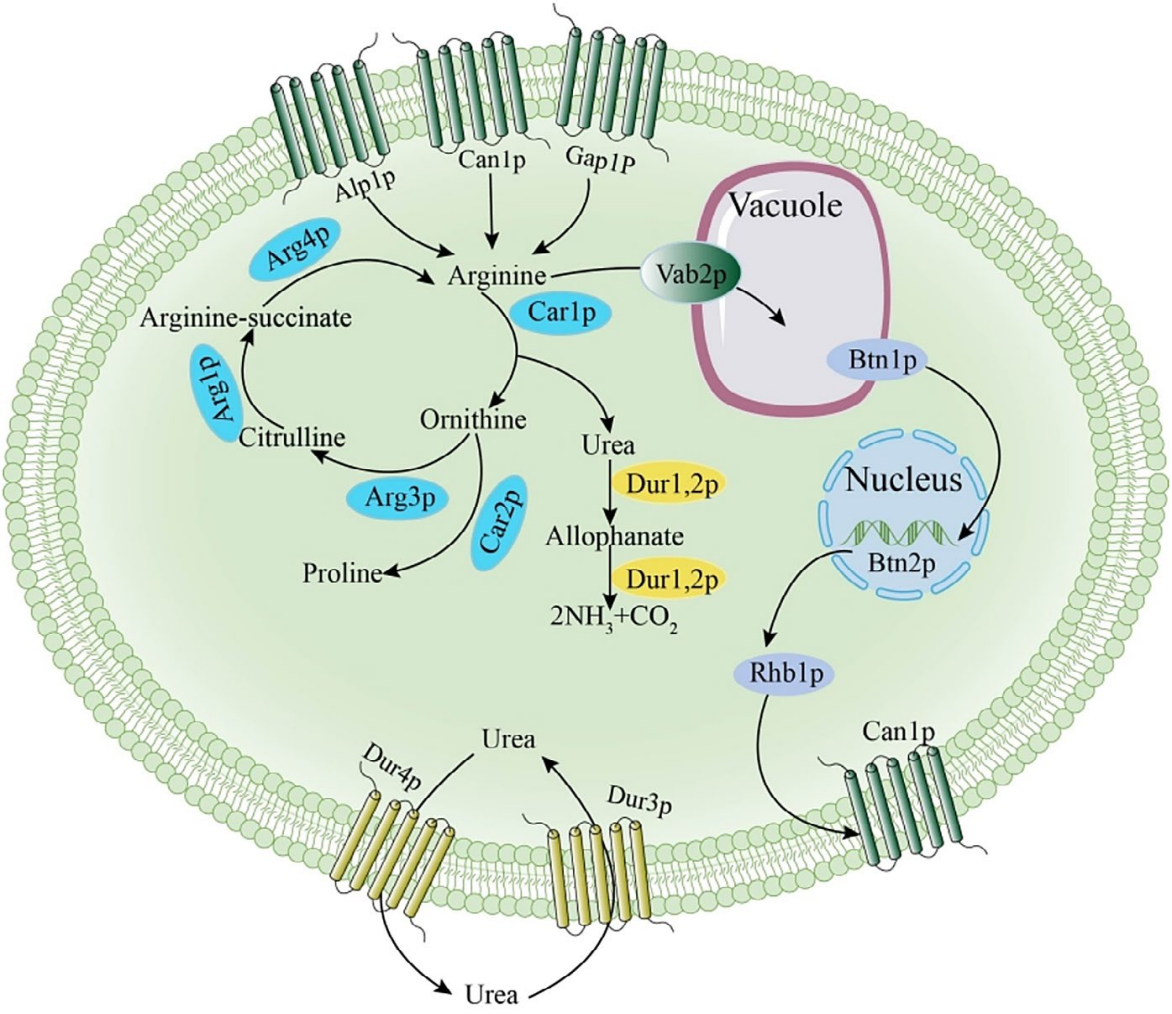
Transportation and metabolism of arginine and urea in *S. cerevisiae* .

The arginine metabolism pathway directly influences the production and elimination of urea. The transportation and metabolism of arginine in *S. cerevisiae* is illustrated in Figure 1. Arginine uptake in the cell membrane was facilitated by multiple transporter proteins, that is, Can1p, Alp1p, and Gap1p. Hoffmann identified Can1p as a specific transporter protein for arginine transport from the extracellular space to the intracellular environment (Hoffmann 1985). Alp1p has been recognized as a potential arginine transporter protein, and the overexpression of *ALP1* significantly enhanced arginine transport (Regenberg et al. 1999). Moreover, Gap1p was a generalized transporter protein that transports all amino acids (Van Zeebroeck et al. 2009). These three transporters regulated one another to maintain a balance of arginine transport (Zhang et al. 2016). After being transported intracellularly, arginine followed two pathways: the vacuolar basic amino acid transporter (Vab2p) transported approximately 90% of arginine into the vacuole, where arginine existed as free arginine (Shimazu et al. 2005; Zhou et al. 2020), playing a crucial role in regulating the expression of genes involved in arginine utilization and inhibiting arginine biosynthesis. The remaining arginine was broken down into urea and ornithine by the arginase (Car1p). Urea was further degraded to CO_2_ and ammonia by urea amidolyase (Dur1,2p) or excreted extracellularly by urea transporter proteins (Dur4p), while ornithine was then converted to proline by ornithine aminotransferase (Car2p) (Ghaddar et al. 2014; Zhang et al. 2016).

A Previous study showed that when *BTN2* was downregulated, the rate of arginine uptake was lowered in the mixed fermentation of *S. cerevisiae* and *L. brevis* , leading to a reduced EC concentration (Fang et al. 2019). Therefore, *BTN2* was considered a potential key regulator of arginine metabolism. Additionally, Btn2p encoded by *BTN2* interacts biochemically and functionally with Rhb1p, which inhibits the activity of arginine permease. *BTN2* knockout failed to localize Rhb1p in the peripheral cellular structures, resulting in reduced arginine uptake by Can1p arginine permease (Chattopadhyay and Pearce 2002; Chattopadhyay et al. 2002). However, the specific effect of *BTN2* on the arginine metabolic pathway and its role in reducing extracellular urea accumulation remain unexplored. Therefore, we utilized *S. cerevisiae* BY4742, *BTN2* knockout, and over‐expressing strains to study arginine metabolites and related transcriptional gene changes, to elucidate the potential mechanisms by which *BTN2* knockout strains reduced arginine uptake, leading to decreased urea accumulation. Meanwhile, *BTN2* was reported to be associated with ethanol tolerance. Under severe ethanol stress, the v‐SNARE binding protein encoded by *BTN2* was involved in intracellular protein transport, played a role in protein deposition in the nucleus (Kato et al. 2018), and was particularly effective in removing denatured proteins caused by severe ethanol stress in yeast (Kato et al. 2019). In this study, we also investigated research on the ethanol tolerance of *BTN2*‐modified strains. The findings revealed the mechanism of arginine transport regulated by *BTN2* and provided a potential application of *BTN2*‐modified strains to reduce urea and EC.

## 2 Materials and Methods

### 2.1 Chemical and Materials

L‐arginine monohydrochloride, L‐ornithine monohydrochloride, urea, diacetyl monoxime, thiosemicarbazide, Malondialdehyde (MDA) Content Assay Kit, and Modified BCA Protein Assay Kit were obtained from Sangon Biotech Corporation (Shanghai, China). 2,4‐dinitrofluorobenzene (DNFB) was purchased from Energy Chemical Corporation (Anhui, China). QuantiChrom Arginase Assay Kit and QuantiChrom Urease Assay Kit were obtained from BioAssay Systems Corporation (Hayward, CA, USA). Yeast RNA Kit R6870 and PrimeScript RT reagent Kit with gDNA Eraser RR047A were purchased from Omega Bio‐Tek (Guangzhou, China) and Takara Bio Inc. (Shiga, Japan), respectively.

### 2.2 Strains and Culture Media

*S. cerevisiae* BY4742 (WT)and *BTN2* over‐expressing strain (*BTN2*↑) were obtained from Hangzhou Medical College; *BTN2* knock out strain (*BTN2*∆) was constructed by Shanghai Jiao Tong university. The yeast strains were cultured on Yeast Extract Peptone Dextrose Medium (YPD): 10 g/L yeast extract, 20 g/L peptone, 20 g/L D‐glucose, 20 g/L agar and rich nitrogen‐derived arginine selective medium SC1, which was composed of YPD medium supplemented with 10 g/L L‐arginine monohydrochloride.

### 2.3 Yeast Cell Growth Curve Assay

BY4742, *BTN2*∆, and *BTN2*↑ cells were cultured in YPD medium until an optical density of 1.0 at OD_600_ (5 × 10^7^ cfu/mL). The cultured cells were then collected, washed, and resuspended with targeted medium. 2 mL of each sample was added to the corresponding 100 mL of medium. Throughout the cultivation period, OD_600_ was recorded every 4 h, and the wet weight of the cells was measured every 12 h.

### 2.4 Arginine Related Metabolites Analysis

Urea concentration was assayed using the described method by Fang et al. 2014. L‐arginine and L‐ornithine were determined by HPLC‐VWD method with an Agilent HPLC system 1260. The derivative reactions were conducted between 150 μL samples (or standard L‐arginine and L‐ornithine) and 150 μL 1% DNFB acetonitrile solution, in which 150 μL NaHCO_3_ (0.5 M) was added before reacting. All the reaction mixtures had to react for 60 min without light and then finalized to 1 mL with PBS buffer (pH 7.0). HPLC conditions were as follows: NaAc‐HOAc buffer (0.02 M, pH 7.4), acetonitrile and water were used as mobile phases A, B, and D. The wavelength was kept at 360 nm, the temperature was set at 30°C, and the injection volume was 20 μL (Wu, Kong, et al. 2020).

### 2.5 Arginase and Urease Activities Analysis

Yeast cells were harvested at logarithmic (8 × 10^7^ cfu/mL), stationary (1 × 10^8^ cfu/mL) and decline (1.1 × 10^8^ cfu/mL) phases by centrifugation at 8000 × g (4°C, 10 min), then washed and resuspended in Tris–HCl buffer (10 Mm pH 7.4). After ultrasonication for 15 min (sonication power: 300 W, duty time: 4 s, interval time: 6 s), the supernatant extract was collected for the following assays. Total protein was determined by the modified BCA method. Determination of arginase and urease activities was performed using the Arginase Assay Kit and urease Assay Kit, respectively. One unit of arginase was defined as the enzyme that converted 1 μmole of L‐arginine to ornithine and urea per minute at pH 9.5 and 37°C, and one unit of urease was defined as the amount of 1 μmol ammonia per min at pH 7.0 under the assay conditions.

### 2.6 Real Time Quantitative PCR (RT‐qPCR) Assay

The RT‐qPCR primers were designed and synthesized by Shanghai Sangong Biological Engineering Corporation. The sequences are shown in Table 1. Total RNA was extracted using Yeast RNA Kit R6870. After DNA removal from the genome, it was held at 42°C for 2 min and then placed at 4°C. cDNA synthesis from total RNA was performed using the PrimeScript RT reagent Kit with gDNA Eraser. The qPCR amplification was performed for a 30 μL sample containing qPCR Mix (15 μL), primer (0.5 μL each) and template DNA (2 μL). All amplifications consisted of an initial amplification at 95°C for 3 min, followed by 40 cycles at 95°C for 15 s, 62°C for 20 s, and 72°C for 20 s. Relative quantification of amplified genes was performed using the 2^−∆∆*Ct*^ method.

**TABLE 1:** Oligonucleotides used for quantitative RT‐PCR.

Gene	Name	Sequence (5′–3′)
*CAR1*	*CAR1*‐F	AATACGGCATCAACGCTGTCATTG
	*CAR1*‐R	CCACCTCTCACTGGAGTACCTGTA
*CAR2*	*CAR2*‐F	GGTATAATCTCTGAGGTGCGTGGTA
	*CAR2*‐R	AAGGAGGAGCCAATCTGATGATGT
*CAN1*	*CAN1*‐F	GGAGGATGGCATAGGTGATGAAGAT
	*CAN1*‐R	GCGTTGGTCAGAGGTGTGGATAA
*ALP1*	*ALP1*‐F	AGTCGAGGATGATGCTGCTAAGGA
	*ALP1*‐R	TGGCGGACCAATACCAATTATGAGG
*GAPL*	*GAP1*‐F	TGGTGGTCCAACAGGTGGTTACAT
	*GAP1*‐R	GCAGCGGTGACGAAGACAGAAC
*DUR1,2*	*DUR1,2*‐F	GGTGTCCCTATTGCTGTTAAG
	*DUR1,2*‐R	CCGTGTGCCGACTAATCC
*DUR3*	*DUR3*‐F	ACTGCCTGTGGGTGTTGTTG
	*DUR3*‐R	CGTCTACTGGATGCCTCTTGG
*ACT1*	*ACT1‐*F	TTATTGATAACGGTTCTGGTATG
	*ACT1*‐R	CCTTGGTGTCTTGGTCTAC

### 2.7 Ethanol Stress Tolerance Assay

Tolerance of yeast strains to various ethanol stresses was compared by spot dilution on solid media (Cheng et al. 2016). After culturing in SC1 medium at 30°C for 48 h, the cell suspensions were collected and diluted serially. 2.5 μL of each 10‐fold dilution (10^−1^–10^−6^) was spotted onto YPD plates added with various concentrations of ethanol (0%, 3%, 6% and 9%, v/v). The yeast cell growth was evaluated after cultivation at 30°C for 72 h with the plates wrapped in sealing film to avoid ethanol evaporation (Morard et al. 2019).

Ethanol tolerance was also assessed using a liquid growth assay (Cheng et al. 2016). Yeast cells were pre‐cultured in YPD (30°C, 220 rpm) for 24 h, transformed into 100 mL of SC1 with ethanol at a final concentration of 0%, 3%, 6%, and 9% (v/v) respectively, and then incubated at 220 rpm, 30°C for the logarithmic phase (16 h and 24 h).

Malondialdehyde (MDA) was also examined to determine the cellular damage caused by ethanol in different yeast strains (Liu et al. 2024). MDA was detected by the MDA Content Assay Kit.

### 2.8 Statistical Analysis

Dates were analyzed using Origin 2021 software and GraphPad Prism 9. The results are presented as mean ± standard deviation (SD) of three determinations. Significant differences between treatments were determined by SPSS (V20.0) software and using Duncan's multiple range test, and statistically significant differences were determined at *p* < 0.5. Asterisks denote significant differences between strains (*p < 0.05; **p < 0.01; ***p < 0.001; ****p < 0.0001).

## 3 Results and Discussion

### 3.1 Effect of the Transcription Factor Btn2p on Cell Growth

**FIGURE 2:**
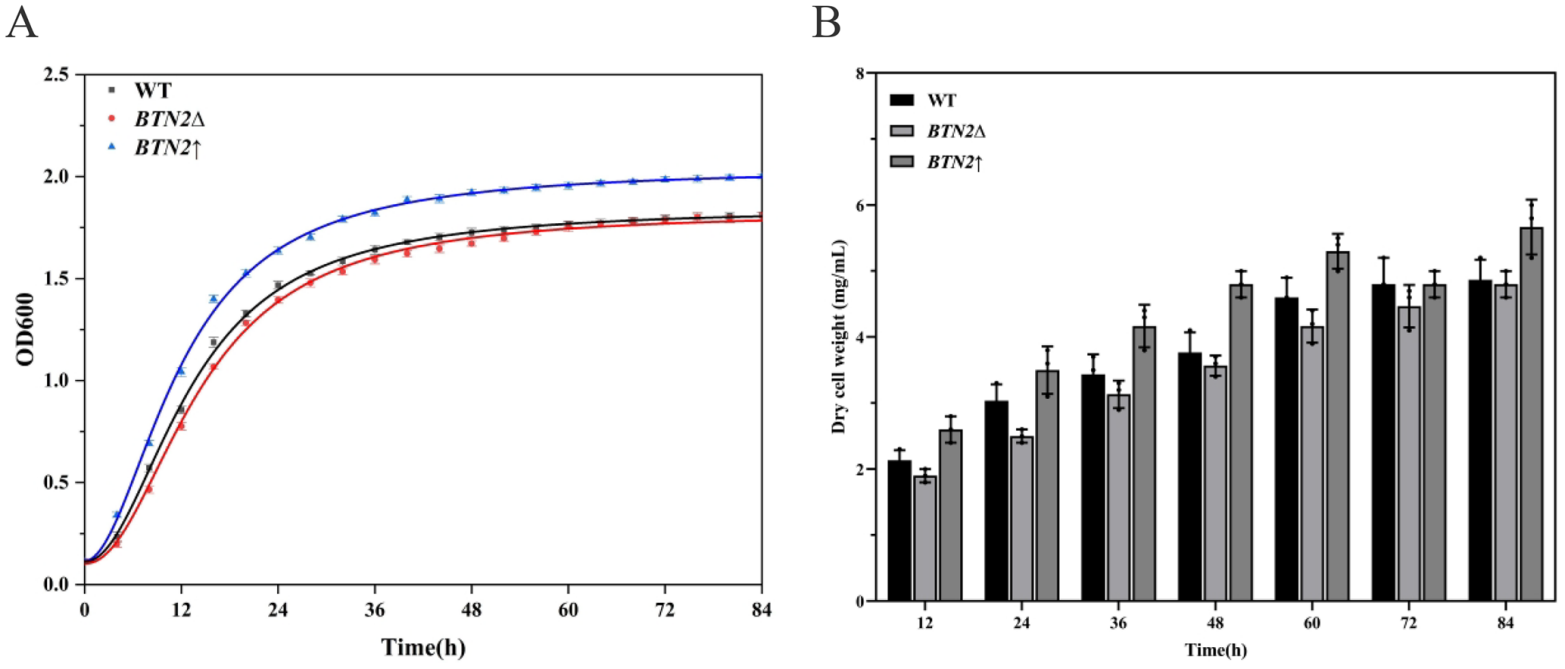
The impact of *BTN2* on *S. cerevisiae* cell growth in YPD medium containing arginine. (A) The growth curves of WT, *BTN2*∆, and *BTN2*↑. (B) Dry cell weight of WT, *BTN2*∆, and *BTN2*↑.

Cell growth curves and dry cell weight were obtained to determine whether *BTN2* affected *S. cerevisiae* growth or not. The data presented in Figure 2 indicated that *BTN2*∆ grew slightly slower than WT. However, there was no significant difference in growth performance or dry cell weight between the *BTN2*∆ and the WT group. Both strains entered the arrest phase at similar periods and displayed comparable logarithmic phase trends. In contrast to the WT, *BTN2*↑ demonstrated superior growth performance with an 11.73% increase in dry cell weight, with a shorter logarithmic growth period and an earlier entry into the stationary phase of growth.

### 3.2 Effect of *BTN2* on Key Substances and Enzyme Activities in Arginine and Urea Metabolism

Arginine, urea, and ornithine are key substances in the metabolism of arginine, in which urea can react spontaneously with ethanol to produce EC (Shalamitskiy et al. 2023). Arginine can be degraded by arginase to produce urea and ornithine, the precursor of citrulline, which can also affect the production of EC (Wei et al. 2020). The consumption of arginine and the accumulation of extracellular urea and ornithine were detected. We selected three specific time points (24, 48 and 72 h) in the logarithmic, stationary, and decline phases to determine key intracellular substances and enzyme activities involved in arginine and urea metabolism.

**FIGURE 3:**
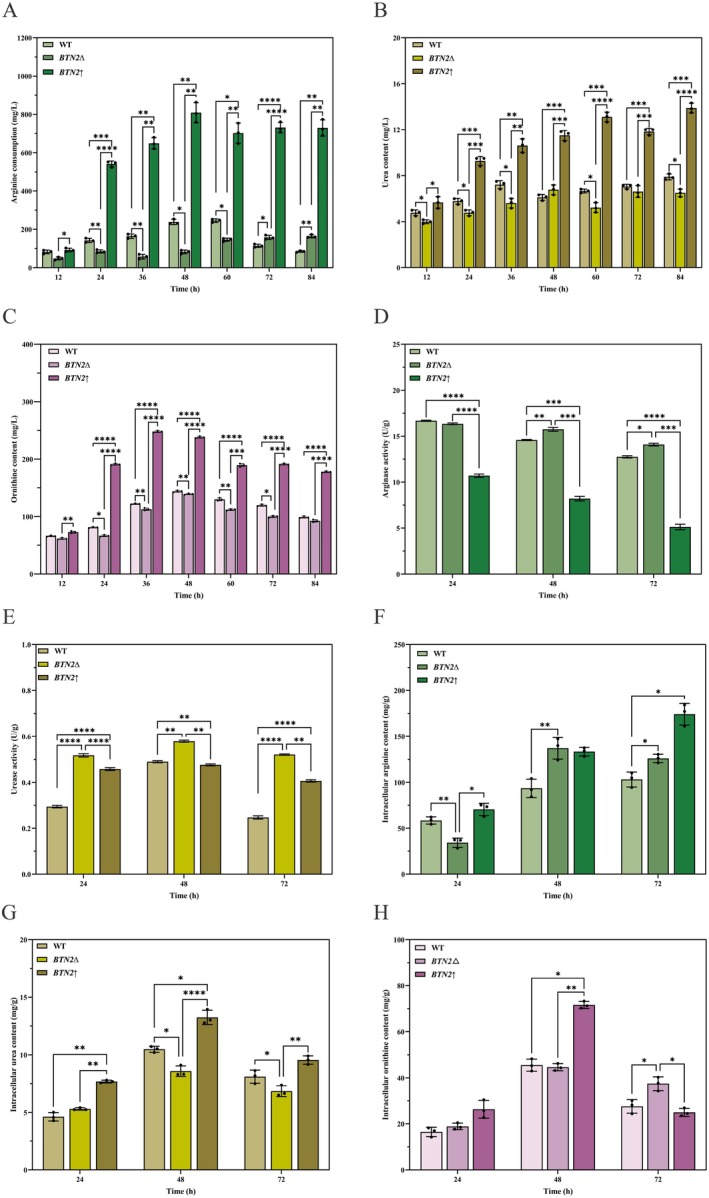
The impact of *BTN2* on arginine and urea metabolism‐related substances and enzyme activities. (A) The arginine consumption in *BTN2‐modified* strains. (B) The urea production in *BTN2‐modified* strains. (C) The ornithine production in *BTN2‐*modified strains. (D) Comparison of arginase activities in *BTN2‐modified* strains. (E) Comparison of urease activities in *BTN2‐modified* strains. (F) Comparison of intracellular arginine content in *BTN2‐modified* strains. (G) Comparison of intracellular urea content in *BTN2‐modified* strains. (H) Comparison of intracellular ornithine content in *BTN2‐modified* strains.

During the cultivation period, the trend of changes in extracellular key substances was consistent among the three strains: *BTN2*↑ > WT > *BTN2*∆ (Figure 3A–C). Extracellular arginine consumption, as well as urea and ornithine content, gradually increased with the prolongation of cultivation time until 48 h. Moreover, a decrease in arginine consumption was observed in WT and *BTN2*↑ after 48 h (Figure 3A), which may be attributed to the release of generated arginine into the extracellular space under the action of arginine synthase during the late growth phase. In contrast, the consumption of arginine in *BTN2*∆ increased consistently, suggesting that *BTN2* knockout may disrupt the function of arginine synthase. At the end of cultivation, urea content of *BTN2*↑ increased by 76.05%, whereas urea content of *BTN2*∆ decreased by 17.62% compared to WT. Interestingly, urea concentration of WT, *BTN2*∆, and *BTN2*↑ gradually reached equilibrium after a decreasing trend over certain cultivation time periods (48, 60, and 72 h). Notably, *BTN2*↑ had significantly higher extracellular key substances levels compared to WT and *BTN2*∆. This might be due to the 11.73% increase in dry cell weight/ml of the constructed *BTN2* over‐expression strain.

In addition, key intracellular substances and enzyme activities were measured at three selected time points (Figure 3D–H). *BTN2*∆ demonstrated a higher arginase activity compared to WT and *BTN2*↑, leading to an increased level of arginine degradation. As a result, *BTN2*∆ exhibited higher intracellular ornithine levels. *BTN2*∆ also showed higher urease activity, resulting in a higher concentration of urea decomposition. Conversely, *BTN2*↑ exhibited lower arginase activity compared to WT. Although sometimes *BTN2*↑ had higher urease activity, a large amount of arginine was transferred from the extracellular to the intracellular environment, resulting in higher intracellular arginine and urea levels.

### 3.3 Transcriptional Analysis of Genes Related to Arginine and Urea Metabolism

**FIGURE 4:**
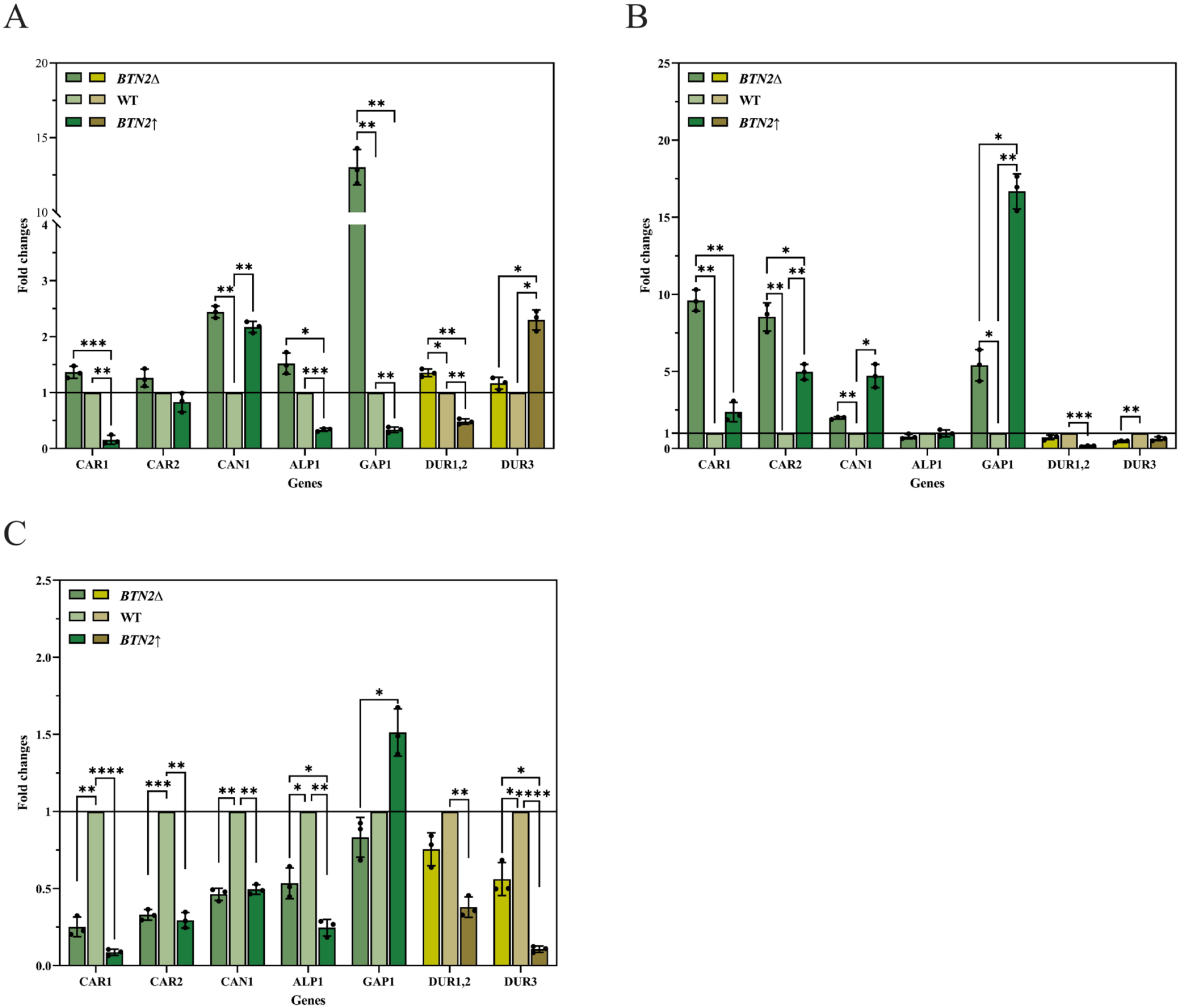
The impact of *BTN2* on the transcript levels of genes related to arginine and urea metabolism. (A) Fold changes of genes related to arginine transport, arginine metabolism, and urea metabolism at 24 h in *BTN2*∆ and *BTN2*↑ compared to WT. (B) Fold changes of genes related to arginine transport, arginine metabolism, and urea metabolism at 48 h in *BTN2*∆ and *BTN2*↑ compared to WT. (C) Fold changes of genes related to arginine transport, arginine metabolism, and urea metabolism at 72 h in *BTN2*∆ and *BTN2*↑ compared to WT.

To further elucidate the correlation between *BTN2* and arginine and urea metabolism, the transcript expression levels of relevant genes in *S. cerevisiae* were assayed by RT‐qPCR at the three selected time points, and the results are shown in Figure 4. At 72 h, compared to WT, all tested genes were down‐regulated except for *GAP1* (1.51‐fold) in *BTN2*↑ (Figure 4C). This indicated that *BTN2* had little transcriptional regulatory effect on arginine and urea catabolism in *S. cerevisiae* during the decline phase, but still influenced arginine transport.

In the logarithmic phase (24 h) (Figure 4A), *BTN2*∆ caused significantly up‐regulated expression of *CAR1* (1.36‐fold), *CAN1* (2.34‐fold), *ALP1* (1.52‐fold), *GAP1* (13.03‐fold), and *DUR1,2* (1.35‐fold). In contrast, B*TN2*↑ led to the down‐regulation of *CAR1* (0.16‐fold), *ALP1* (0.34‐fold), *GAP1* (0.34‐fold), and *DUR1,2* (0.48‐fold) expression. In the stationary phase (48 h) (Figure 3B), both *BTN2*∆ and *BTN2*↑ triggered the up‐regulation of *CAR1*, *CAR2*, *CAN1*, and *GAP1*. Therefore, it could be assumed that the yeast may have taken up arginine during the stationary phase, leading to the simultaneous up‐regulation of *CAR1* and *CAR2* in *BTN2*‐modified strains. Furthermore, the complex composition of the YPD medium, which contains various amino acids, along with the mutual regulation of *CAN1*, *GAP1*, and *ALP1*, resulted in the concurrent up‐regulation of *CAN1* and *GAP1* in the *BTN2*‐modified strains. However, the impact of *BTN2* on arginine metabolism could still be assessed by examining the fold‐change in upregulation. Notably, *BTN2*↑ significantly increased the expression of *CAN1* (4.97‐fold) and *GAP1* (16.67‐fold). These findings indicate that *BTN2* may affect arginine metabolism in *S. cerevisiae* by affecting arginine transport during growth.

From the logarithmic phase (24 h) to the stationary phase (48 h), the expression of *CAR1* and *CAR2* in *BTN2*∆ consistently showed up‐regulation while folds reduced from 13.08 to 5.41 in *GAP1* and from 2.44 to 1.98 in *CAN1*. Alkim et al. (2013) also discovered that in cobalt stress‐tolerant *S. cerevisiae* , *BTN2* was downregulated, whereas *CAR1* was upregulated. Until the decline phase (72 h), *GAP1* and *CAN1* showed down‐regulation. Conversely, the expression of *CAN1* (4.97‐fold) and *GAP1* (16.67‐fold) in *BTN2*↑ exhibited a significant increase, and *GAP1* was still up‐regulated until the decline phase. These results indicated that *BTN2* influences *CAN1* and *GAP1*. Herein, it could be assumed that knocking out *BTN2* would reduce arginine uptake in *S. cerevisiae* and ultimately decrease the extracellular urea concentration.

### 3.4 Effects of *BTN2* ‐Modified Strains Under Ethanol Stress on *S. cerevisiae*

**FIGURE 5:**
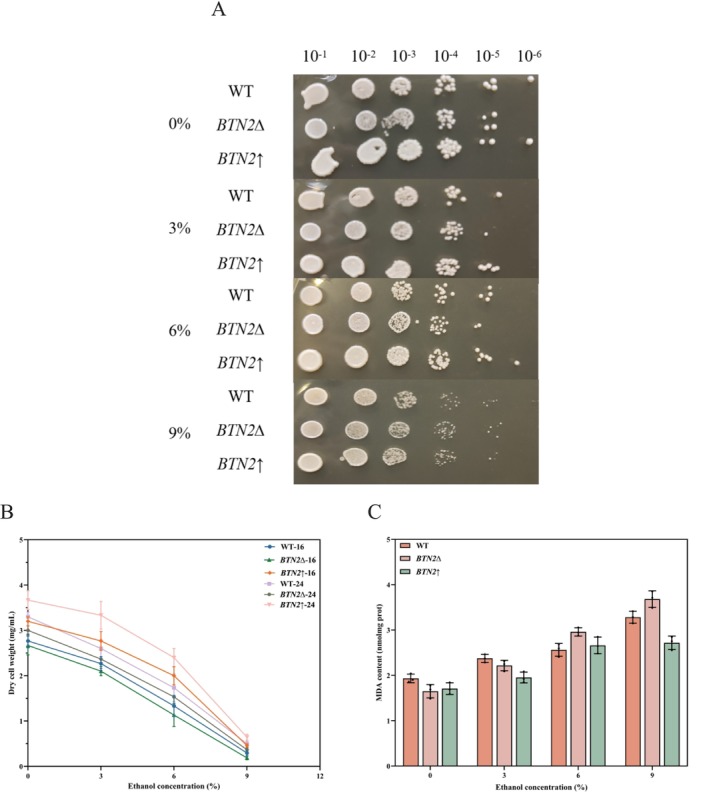
Response of *BTN2* modified strains to ethanol stress. (A) Growth of *BTN2* modified strains on YPD solid media under various ethanol stresses by spot dilution assay. (B) Comparison of the growth of *BTN2* modified strains at 16 h and 24 h in cultures under various ethanol stresses by dry cell weight assay. (C) Comparison of the degree of lipid peroxidation of cell membranes of *BTN2* modified strains in cultures under various ethanol stresses by MDA assay.

Espinazo‐Romeu et al. (2008) found that Btn2p is important for amino acid transport and for ethanol resistance. For further application of *BTN2* modified strains in Chinese rice wine fermentation, their tolerance to ethanol was carefully investigated. In a study of an evolved *S. cerevisiae* strain tolerant to oxidative stress, Kocaefe‐Ozsen et al. (2022) reported ethanol tolerance and upregulation of *BTN2* compared to the original reference strain. Growth of the three strains was compared in solid YPD medium at different ethanol concentrations, as shown in Figure 5A. No significant differences in cell growth were observed at low ethanol concentrations (3% and 6%); however, the growth of these cells was strongly inhibited under severe ethanol stress (9%). Notably, the growth status of the three strains was *BTN2*↑ > WT > *BTN2*∆.

To validate the spot assay results, we carried out liquid cultivations with various ethanol concentrations. Comparing the dry cell weight at 16 and 24 h (Figure 5B), the three strains showed varying degrees of ethanol tolerance, with the most significant inhibition observed at 9% ethanol concentrations. After being cultivated with 9% ethanol for 24 h, WT, *BTN2*∆, and *BTN2*↑ cell densities decreased by 84.55%, 89.67%, and 78.74%, respectively, compared with non‐stressed conditions. In further cell damage assays, the degree of cell membrane peroxidation at 24 h was determined using MDA (Figure 5C). Under 9% ethanol stress, MDA levels increased 1.70, 2.23, and 1.59 times in WT, *BTN2*∆, and *BTN2*↑, respectively, compared with 0% ethanol conditions. Ethanol increased MDA content, indicating ethanol‐induced oxidative stress in the cells. These results showed that *BTN2* enhanced the cell's ethanol tolerance and membrane integrity while decreasing ethanol‐induced oxidative damage.

## 4 Conclusion

In conclusion, *BTN2* significantly affects arginine and urea metabolism in *S. cerevisiae* . It regulates arginine transportation, catabolism, and urea degradation by modulating the expression of arginine transporter proteins (*GAP1* and *CAN1*), arginase (*CAR1*), and urea amidolyase (*DUR1,2*). *BTN2* knockdown has demonstrated positive effects on the reduction of arginine uptake and urea accumulation. Although *BTN2*∆ reduced arginine uptake, intracellular arginine levels remained high after cell fragmentation, indicating that *BTN2*∆ may increase arginine uptake by vacuole. This, in turn, could inhibit arginine metabolism, thereby suppressing extracellular urea accumulation as well. On the contrary, *BTN2*∆ enhanced urea catabolism, leading to decreased extracellular urea accumulation. Besides, we found that *BTN2* was associated with ethanol tolerance and it could reduce ethanol damage to cell membranes. Therefore, *BTN2* is thought to be a key regulator of arginine and urea metabolism in *S. cerevisiae* and may be used as a potential target for EC reduction in Chinese rice wine fermentation.

## Author Contributions

**Ting Xia:** data curation (equal), software (equal), writing – original draft (lead), writing – review and editing (equal). **Keiwei Chen:** methodology (equal). **Huqi Zhou:** supervision (equal), validation (equal). **Tangchao Chen:** supervision (equal). **Wenjing Lin:** formal analysis (equal). **Gongnian Xiao:** resources (equal). **Ruosi Fang:** funding acquisition (equal), project administration (equal), resources (equal), writing – review and editing (equal).

## Conflicts of Interest

The authors declare no conflicts of interest.

## Data Availability

All data generated or analyzed during this study are included in this article. Further inquiries can be directed to the corresponding author.
